# Can routinely collected administrative data effectively be used to evaluate and validate endpoints used in breast cancer clinical trials? Protocol for a scoping review of the literature

**DOI:** 10.1186/s13643-023-02283-5

**Published:** 2023-07-08

**Authors:** Hely Shah, Dianna Wolfe, Mark Clemons, Michelle Liu, Kednapa Thavorn, Areti-Angeliki Veroniki, Carole Lunny, Greg Pond, Sharon McGee, Becky Skidmore, Angel Arnaout, Brian Hutton

**Affiliations:** 1 Department of Oncology, Ottawa Hospital, Ottawa, ON Canada; 2 Ottawa Hospital Research Institute, Ottawa, ON Canada; 3 Knowledge Translation Program, Li Ka Shing Knowledge Institute, St. Michael’s Hospital, Toronto, ON Canada; 4 Institute for Health Policy, Management, and Evaluation, University of Toronto, Toronto, ON Canada; 5 Department of Oncology, McMaster University, Hamilton, ON Canada

**Keywords:** Breast cancer, Recurrence, Administrative data, Real-world data, Scoping review

## Abstract

**Background:**

Randomized controlled trials (RCTs) are a critical component of evidence-based medicine and the evolution of patient care. However, the costs of conducting a RCT can be prohibitive. A promising approach toward reduction of costs and lessening of the burden of intensive and lengthy patient follow-up is the use of routinely collected healthcare data (RCHD), commonly called real-world data. We propose a scoping review to identify existing RCHD case definitions of breast cancer progression and survival and their diagnostic performance.

**Methods:**

We will search MEDLINE, EMBASE, and CINAHL to identify primary studies of women with either early-stage or metastatic breast cancer, managed with established therapies, that evaluated the diagnostic accuracy of one or more RCHD-based case definitions or algorithms of disease progression (i.e., recurrence, progression-free survival, disease-free survival, or invasive disease-free survival) or survival (i.e., breast-cancer-free survival or overall survival) compared with a reference standard measure (e.g., chart review or a clinical trial dataset). Study characteristics and descriptions of algorithms will be extracted along with measures of the diagnostic accuracy of each algorithm (e.g., sensitivity, specificity, positive predictive value, negative predictive value), which will be summarized both descriptively and in structured figures/tables.

**Discussion:**

Findings from this scoping review will be clinically meaningful for breast cancer researchers globally. Identification of feasible and accurate strategies to measure patient-important outcomes will potentially reduce RCT budgets as well as lessen the burden of intensive trial follow-up on patients.

**Systematic review registration:**

Open Science Framework (https://doi.org/10.17605/OSF.IO/6D9RS)

## Background

Significant advances have been made in the treatment of early-stage breast cancer through the performance of large, randomised controlled trials (RCTs). However, traditional, large RCTs comparing adjuvant interventions are challenging and expensive to conduct, with the average per-study cost for a Phase III US oncology trial estimated to be $22.1 million USD [1]. Historically in breast cancer trials, the gold standard for collecting well established efficacy endpoints [2] related to disease progression, including recurrence, disease-free survival (DFS), invasive disease-free survival (iDFS), overall survival (OS), progression-free survival (PFS), and invasive breast cancer-free survival (iDFS) has been prospective, individual patient follow-up. However, low occurrence rates of these outcomes often result in limited numbers of events over long follow-up periods, high financial costs of study staff and testing regimens, and significant burdens on trial participants. Novel trial designs may reduce some of these challenges and the expense of intensive individual patient follow-up. One potential strategy is the exploitation of routinely collected healthcare data (e.g., administrative data) from one or more source databases [3–7].

Routinely collected healthcare data (RCHD; or real-world data) are data that have been systematically collected for reasons other than research or without specific research questions. Examples of RCHD include information from electronic health records (EHRs), health administration data, disease registries, and epidemiologic surveillance systems [6]. Administrative data have been shown to be a compelling source of long-term comparative effectiveness data in registry based RCTs, demonstrating minimal losses to follow-up for rare outcomes that require long follow-up periods [8]. In cardiovascular research, investigators have used RCHD by integrating administrative data and EHRs to create a cardiovascular-specific database that supports data analytics in their field [9]. Similarly, harmonized data sets such as the administrative datasets housed at the Institute for Clinical Evaluative Sciences (ICES) in Ontario, Canada, have been shown to have the potential to improve the economy and quality of data collected in clinical trials, while minimizing data collection burdens on patients [10, 11]. Furthermore, with patient consent, prospective linkage of personal data with health administrative records was both feasible and accurate [11, 12]. Another study of the utility of health administrative data to identify breast cancer recurrence in reproductive-aged women found that recurrence could be detected with moderate validity using a case definition of greater than or equal to 10 months between original diagnosis date and the subsequent appearance of two or more cancer diagnosis codes [13]. The validity of detection of breast cancer recurrence in administrative datasets may be further improved using computer-coded algorithms of high sensitivity and specificity.

Breast cancer recurrence is not directly captured in RCHD sources with a diagnostic code [14], such as an ICD-9/10 code, and instead must be inferred from the accumulation of other diagnostic codes and health system contacts made by the patient during the investigation of potential disease progression. The presence of diagnostic codes—including additional diagnoses, laboratory tests, imaging evaluations, and drug prescriptions—in a patient’s electronic health information and their timing relative to the initial breast cancer diagnosis may be analysed to identify patterns indicative of incident disease progression. Detection of such codes or patterns can identify a patient for follow-up by the study team to confirm if a disease progression event has occurred. The targeted follow-up of only trial participants that likely have experienced disease progression reduces the high costs and burdens of scheduled follow-up at regular intervals required of all RCT participants in order to collect data on both disease progression and survival. Additionally, case definitions for disease progression based upon diagnostic codes in RCHD can be used to inform outcome identification for the purposes of data analysis. This strategy can also reduce costs and provide an advantageous source of long-term follow-up information.

The use of RCHD also has limitations [10, 11, 15, 16]. Generally, only quantitative data are available for specific outcomes, such as survival or hospital visits, which limits the scope of research objectives that can be addressed. Qualitative health behaviours and other endpoints of importance in oncology studies, such as the occurrence of and date of disease recurrence for calculations of DFS or PFS, cannot be routinely analysed. Systematic reviews of the use of administrative data for non-cancer conditions such as sepsis, heart failure, and neurologic conditions have shown that the diagnostic performance of endpoint detection algorithms can vary notably in relation to the number of codes used [17–19]. We will extend previous review strategies to breast cancer by conducting a scoping review to map the features and diagnostic performance of existing case definitions and algorithms using RCHD that have been used to define recurrence, DFS, iDFS, OS, and breast-cancer-free survival (BCFS) in early-stage breast cancer patients (i.e., neo/adjuvant patients), as well as PFS and OS in metastatic breast cancer patients.

## Methods

This scoping review will be performed in consideration of methods guidance from JBI (formerly the Joanna Briggs Institute) [20, 21]. This protocol has been registered with the Open Science Framework (https://doi.org/10.17605/OSF.IO/6D9RS); given the iterative nature of scoping reviews, protocol amendments with their rationale will be documented in the completed review. This protocol has been reported with consideration of the Preferred Reporting Items for Systematic Review and Meta-Analysis Extension Statement for Protocols (PRISMA-P) [22, 23] and reporting of the final review will be guided by the PRISMA Extension Statement for Scoping Reviews (PRISMA-ScR) [24]. We will address the following review questions:

1. What existing case definitions in terms of diagnostic and billing codes within RCHD sources, their timing relative to original breast cancer diagnosis, or other features have been studied to identify disease progression and survival events in breast cancer patients?
2. What was the diagnostic performance of these case definitions compared to a reference standard, as measured by sensitivity, specificity, positive predictive value (PPV), negative predictive value (NPV) and/or other measures?

### Study eligibility criteria

We will use the following selection criteria to identify relevant studies for the planned review, guided by the Population – Concept – Context (PCC) framework.

#### Population

Women with either early-stage or metastatic breast cancer managed with established breast cancer therapies (e.g., chemotherapy, radiation, repeat surgery). Sample of mixed cancer populations (e.g., breast cancer and colon cancer) will be excluded unless separate findings have been reported specific to breast cancer.

#### Concept

Primary studies examining the diagnostic accuracy of one or more case definitions or algorithms of disease progression (i.e., recurrence, PFS, DFS, or iDFS) or survival (i.e., BCFS or OS) compared with a reference standard measure (e.g., chart review, clinical trial dataset). Diagnostic accuracy is anticipated to be reported as sensitivity, specificity, PPV, NPV, or an estimate of area under the curve (AUC), however other measures of agreement will also be considered. Case definitions and algorithms must have been applied to RCHD/administrative data sources or EHRs, with the goal of detection or estimation of time of occurrence of one or more of the progression or survival events above. Studies involving the use of machine learning methods such as natural language processing to process unstructured data (e.g., clinician notes from electronic health records) for use in case definitions will also be of interest. Algorithms or models developed to predict future survival or another endpoint will be excluded. Studies focusing on differences in algorithm diagnostic accuracy with different data sources will be excluded.

#### Context

Studies from any geographic region will be of interest. Only studies published in English will be sought, without restriction on date of publication.

### Information sources and searching the literature

Literature search strategies will be developed for MEDLINE, EMBASE, and CINAHL using controlled vocabulary (e.g., MEDLINE subject headings) and free-text words by an experienced information specialist with input from the project team (see Appendix). A second information specialist will peer review the strategies using the Peer Review of Electronic Search Strategies (PRESS) Checklist [25]. Searches will be restricted to the English language and animal records will be removed.

### Processes for study selection

Records will be downloaded and deduplicated using EndNote version 9.3.3 (Clarivate Analytics) and uploaded to the online systematic review software DistillerSR^®^ (Evidence Partners Inc, Ottawa, Canada). Screening of citations will be conducted by two independent reviewers first using titles and abstracts (Stage 1 screen). The full texts of the potentially relevant citations identified at Stage 1 will be further screened by two independent reviewers (Stage 2 screen). A calibration exercise will precede both stages of screening to ensure consistency in the application of eligibility criteria by reviewers (batches of 50 to 100 citations at Stage 1 and batches of five full texts at Stage 2, until conflicts are less than 5% and all reviewers are comfortable with the screening criteria). Conflicts during screening will be resolved by discussion until consensus is reached or by consultation with a third review team member. In the final review report, we will document the study selection process using a PRISMA flow diagram and include a list of studies excluded at Stage 2 screening, with reasons for exclusion [26].

#### Use of artificial intelligence – stage 1 screening

The artificial intelligence/machine learning (AI/ML) feature of DistillerSR^®^ will be used to perform prioritized Stage 1 screening [27]. The AI/ML algorithm will be trained by the reviewers, who will begin by screening a small number of known relevant citations along with an additional random sample of citations from the search results to a total of 200 citations. This will expose the AI/ML tool to both relevant and non-relevant citations. The AI/ML tool will subsequently generate relevance scores for the remaining citations (i.e., an estimate of the probability of relevance), which will be used to order the citations from high to low potential relevance as they are presented to the review team for screening. The tool will continue to learn and re-order citations throughout Stage 1 screening. The study team will monitor and resolve conflicts frequently throughout Stage 1 screening to ensure the AI/ML tool continues to be trained on accurate selection decisions. The study team will monitor the proportion of predicted relevant references that have been found (a measure approximated by the AI/ML tool) as well as the decline in new relevant citations identified over time. Once 95% of predicted relevant references have been identified and the yield of new relevant citations is minimal, the AI/ML tool will be used as a single reviewer to exclude all remaining unscreened citations. A single human reviewer will continue to screen all citations and will re-engage a second human team member at any time there is a disagreement with the AI/ML screener. This process will allow for efficiencies in Stage 1 screening, while ensuring two reviewers can still be involved as needed to minimize the risk of omissions related to use of the AI/ML tool. Members of the study team (BH, DW) have several years of experience in the use of DistillerSR’s AI/ML tool and will lead its implementation in this scoping review.

### Data collection

Once all relevant studies have been identified, data extraction will be performed by two reviewers using a standardized extraction form in DistillerSR® software. A pilot extraction exercise will first be performed on a selection of three studies to ensure consistency between reviewers. Data collection will consist of gathering the following information from each included publication: *study characteristics* (e.g., authors, year/journal of publication, country of study performance, breast cancer population [early-stage versus metastatic]), *treatment characteristics* (e.g. endocrine therapy, chemotherapy, biological-targeted therapies), *data source characteristics* (e.g. name, location, type), *study methods* (e.g., description of algorithms/case definitions assessed, data linkage information, type of reference standard group, years of data studied, description of enrolment criteria of study population, statistical methods used to assess performance characteristics), *data summaries related to diagnostic accuracy of each algorithm evaluated* (e.g., sensitivity, specificity, PPV, NPV, or related data if these measures are not reported, but information to inform their calculation are available), and a summary of authors’ cited limitations and conclusions. No risk of bias appraisals will be performed, in alignment with common practice for scoping reviews [21].

### Data analysis

Given the chosen scoping review design and data types extracted, we will employ a descriptive approach to synthesis to summarize the methods and findings of the included studies, supplemented by use of tables and figures to convey key data. Presentation of results will be stratified by outcome. We will use tables to present information regarding study populations (i.e., key clinical features, years of study data and geographic setting), study design characteristics (e.g., nature of reference standard, study size, relevant information regarding data linkages), and details regarding the algorithm employed to assess the outcome(s) of interest (e.g., ICD codes used and other pertinent information). Diagnostic accuracy for each of the algorithms or case definitions used per outcome (i.e., sensitivity, specificity, PPV, NPV) will be summarized both descriptively and in structured figures/tables as determined to be most intuitive by the research team. Comparisons with clinical trial data (if discussed) and study limitations will be similarly summarized. Quantitative results will also be described in tables and figures.

## Discussion

Findings from this scoping review will be clinically meaningful for breast cancer researchers. Our research team, the RE-thinking Clinical Trials Program (REaCT; https://react.ohri.ca), is Canada’s largest pragmatic oncology trials program based in Ontario, Canada. In addition to performing pragmatic trials, the REaCT mandate is to identify feasible and accurate strategies to measure patient-important outcomes in ways that lessen burden for patients. To date this has included strategies such as implementing oral consent, avoidance of trial-mandated clinic visits and the use of virtual visit techniques to make trial participation available for patients irrespective of how far they live from a cancer centre [28]. Despite these strategies, long-term follow-up of patients remains a costly component of performing clinical trials and can be cost prohibitive for obtaining peer-reviewed funding for innovative studies that could significantly improve the care of cancer patients. Hence, if the using real-world data enables patients to be reliably followed for various clinical trial endpoints, it could provide a paradigm shift that will reduce study budgets and make study participation easier for patients and their families. Such benefit would be a major improvement both in Canada and globally.

## Data Availability

Not applicable.

## Appendix

### Search strategy

#### Ovid MEDLINE(R) ALL - search strategy

--------------------------------------------------------------------------------

1 exp Breast Neoplasms/ (328438)

2 ((breast* or mamma or mammar*) adj3 (cancer* or carcinoid* or carcinoma* or carcinogen* or adenocarcinoma* or adeno-carcinoma* or malignan* or neoplasia* or neoplasm* or sarcoma* or tumour* or tumor*)).tw,kw,kf. (400011)

3 1 or 2 [BREAST CANCER] (461908)

4 “International Classification of Diseases”/ (9120)

5 ((international classification adj2 disease?) or ICD or ICD9* or ICD-9* or ICD10* or ICD-10* or ICD11 or ICD-11*).tw,kw,kf. (53024)

6 “Datasets as Topic”/ (7262)

7 “Databases as Topic”/ (9689)

8 “Databases, Factual”/ (95378)

9 (data or database* or data base* or databank* or data bank* or dataset? or data set? or data warehouse?).ti,kw,kf. (338996)

10 ((admin* or billing* or claim? or diagnos* or discharg* or factual or hospital* or insurance or link* or managed care or patient* or utili#ation) adj3 (data or database* or data base* or databank* or data bank* or dataset? or data set? or data warehouse?)).tw,kw,kf. (327596)

11 exp Medical Records/ (156584)

12 Hospital Records/ (3391)

13 Records/ (6443)

14 (chart* or code or coded or codes or coding* or record?).ti,kw,kf. (125184)

15 ((admin* or billing* or claim? or clinical or diagnos* or discharg* or health or health care or healthcare or hospital* or insurance or link* or managed care or medical* or patient* or utili#ation*) adj3 (chart* or code or coded or codes or coding* or record?)).tw,kw,kf. (295753)

16 ((EHR or EMR or EPR) adj10 (electronic* or record*)).tw,kw,kf. (11051)

17 ((admin* or billing* or claim? or diagnos* or discharg* or hospital* or insurance or link* or managed care or utili#ation) adj3 (data or database* or data base* or databank* or data bank* or dataset? or data set? or data warehouse?)).tw,kw,kf. (140677)

18 Registries/ (104981)

19 (register? or register-based or registry* or registries*).tw,kw,kf. (239758)

20 (eregister* or e-register* or eregistr* or e-registr*).tw,kw,kf. (114)

21 (rRCT or rRCTs).tw,kw,kf. (20)

22 Classification/ (10588)

23 (classify* or classification* or misclassify* or mis-classify* or misclassification* or mis-classification*).tw,kw,kf. (456876)

24 case definition*.tw,kw,kf. (6615)

25 Routinely Collected Health Data/ (74)

26 (routine* adj3 (data or database* or data base* or databank* or data bank* or dataset? or data set? or data warehouse?)).tw,kw,kf. (13111)

27 (real-world adj3 (data or database* or data base* or databank* or data bank* or dataset? or data set? or data warehouse?)).tw,kw,kf. (11814)

28 ((RCHD or RWD) adj10 (real or routine* or data)).tw,kw,kf. (333)

29 Health Information Systems/ (1549)

30 Health Surveys/ (66301)

31 *“Surveys and Questionnaires”/ (49904)

32 survey*.ti,kw,kf. (190635)

33 Population Surveillance/ (62371)

34 surveillance*.ti,kw,kf. (61469)

35 ((epidemiolog* or population* or disease* or breast or cancer* or carcinoid* or carcinoma* or carcinogen* or adenocarcinoma* or adeno-carcinoma* or malignan* or neoplasia* or neoplasm* or sarcoma* or tumour* or tumor*) adj3 (survey* or surveillance*)).tw,kw,kf. (89183)

36 “Surveillance, Epidemiology, and End Results”.tw,kw,kf. (12432)

37 SEER.tw,kw,kf. (10433)

38 or/4-37 [DATA/RECORDS/REGISTRIES ETC] (2154144)

39 3 and 38 [BREAST CANCER - DATA/RECORDS/REGISTRIES ETC] (47801)

40 Validation Study.pt. (109068)

41 Validation Studies as Topic/ (2394)

42 valid*.tw,kw,kf. (905538)

43 exp “Reproducibility of Results”/ (450043)

44 reproducib*.tw,kw,kf. (182545)

45 reliab*.tw,kw,kf. (562582)

46 exp Data Collection/st [standards] (45243)

47 “Datasets as Topic”/st [standards] (225)

48 “Databases as Topic”/st [standards] (232)

49 “Databases, Factual”/st [standards] (2081)

50 “International Classification of Diseases”/st [standards] (653)

51 exp Records/st [standards] (15423)

52 Data Accuracy/ (3604)

53 (accura* adj3 (data or database* or data base* or databank* or data bank* or dataset? or data set? or data warehouse?)).tw,kw,kf. (19842)

54 (accura* adj3 (chart* or code or coded or codes or coding* or record?)).tw,kw,kf. (3129)

55 (accura* adj3 classif*).tw,kw,kf. (22721)

56 (accura* adj3 ICD*).tw,kw,kf. (238)

57 exp “Forms and Records Control”/ (10170)

58 ((defin* or gold or reference) adj standard*).tw,kw,kf. (104286)

59 ascertain*.tw,kw,kf. (103232)

60 Algorithms/ (289567)

61 algorithm*.tw,kw,kf. (318830)

62 ((chart* or note* or record*) adj3 (audit* or review*)).tw,kw,kf. (129096)

63 (crosscheck* or cross-check*).tw,kw,kf. (2444)

64 agreement*.ti,kw,kf. (13086)

65 agree*.ab. /freq = 2 (84904)

66 concordan*.tw,kw,kf. (78278)

67 or/40-66 [VALIDITY] (2482105)

68 39 and 67 [BREAST CANCER - RECORDS/DATA/REGISTRIES ETC - VALIDITY] (12586)

69 exp Animals/ not Humans/ (5023939)

70 68 not 69 [ANIMAL-ONLY REMOVED] (12536)

71 limit 70 to english (12198) [LANGUAGE LIMIT APPLIED]

72 exp Breast Neoplasms/mo [mortality] (24987)

73 Mortality/ (48915)

74 Fatal Outcome/ (66244)

75 (fatalit* or mortalit* or death*).tw,kw,kf. (1691608)

76 Recurrence/ (195320)

77 Neoplasm Recurrence, Local/ (137394)

78 (recur* or recrudescenc* or relaps* or recidive* or secondar* or relaps* or progressive or progression* or exacerbat* or advanc* or deteriorat*).tw,kw,kf. (3419392)

79 Survival/ (4909)

80 Disease-Free Survival/ (80595)

81 Progression-Free Survival/ (7735)

82 Survival Analysis/ (145011)

83 surviv*.tw,kw,kf. (1327137)

84 (DFS or iDFS).tw,kw,kf. (21220)

85 exp Disease Progression/ (203039)

86 or/72-85 [OUTCOMES OF INTEREST] (5602390)

87 71 and 86 (5067) [BREAST CANCER - RECORDS/DATA/REGISTRIES ETC – OUTCOMES – VALIDITY]

## Abbreviations

AI/ML: Artificial intelligence/Machine learning
AUC: Area under the curve
BCFS: Breast cancer-free survival
DFS: Disease-free survival
EHR: Electronic health record
ICD: International Classification of Diseases
ICES: Institute for Clinical Evaluative Sciences
iDFS: Invasive disease-free survival
NPV: Negative predictive value
OS: Overall survival
PCC: Population-concept-context framework
PFS: Progression-free survival
PPV: Positive predictive value
PRESS: Peer Review of Electronic Search Strategies
PRISMA: Preferred Reporting Items for Systematic Review and Meta-analysis
RCHD: Routinely collected healthcare data
RCT: Randomised controlled trial
USD: United States dollars
